# The “Devitalization of Pulps.”—A Confession

**Published:** 1894-02

**Authors:** C. M. Weight

**Affiliations:** Cincinnati


### The "Devitalization of Pulps."—A Confession.

BY C. M. WRIGHT, D. D. S., CINCINNATI.

Read before the Ohio State Dental Society, December, 1893.

Having been actively engaged in the practice of dentistry for thirty years, I want to confess that during all this time, I have had trouble and annoyance with the little organs of nutrition which nature, in some freak, developed in the central portions of the teeth. The organs which we classify anatomically, as *pulps* of teeth. Histology has taught us what tissues go to make up these bodies. Physiology tries to satisfy us that their function is a useful one. Embryology has interested us in discussions about their development. Pathology proves to us they are liable to various structural and functional disorders. And Etiology has pointed quite plainly to the causes of the variations from a normal or physiological condition; but with all the knowledge thus offered by these important "ologies"—therapeutics seems sadly at fault, and offers meager means of cure and seemingly unscientific methods. Therapeutics, when applied to disordered pulps, is weak and not progressive.

Thirty years ago we applied arsenious acid, ground up with creasote, sulphate of morphia, charcoal, oil of cloves, etc., etc., in quantities of about one-fiftieth of a grain to "exposed pulps"—and covered the same with cotton and sandarac or gutta-percha Thirty years ago the patient left the office of the dentist with the assurance that "possibly there may be a little pain for a while, but the nerve will be killed," and in a quarter of an hour the little pain began, and for hours it increased in size, while the patient raved and swore and knocked himself about, till he returned to the same dentist, or to another more accessible, with blood-shot eyes, and agonizing throbs over the entire side of the face and head, to beg for the immediate extraction of the tooth. The dentist did not comply then any easier than he does to-day, but with soothing words and expressions of astonishment at the unusual conduct of this particular case under the application, and fair promises of relief, he removed the plug and the arsenical preparation, washed out the cavity with warm water, pricked the congested pulp until hemorrhage ensued, applied some opium or creasote, or clove oil, until the pain became less and dismissed the patient for a day or two.

The same mild fiction with the same tragical environment is constantly repeated to-day. In the meantime many "sure cure' methods recommended by speakers in dental societies, writers for dental journals, and friends in consultation have been faithfully tried.

1. Cobalt has been substituted for arsenious acid.

2. Pressure of the medicament as a cause of pain instead of the arsenious acid or cobalt has been deeply considered, and saucer-shaped tin disks with the medicine in a comparatively fluid condition have been cautiously placed over the exposed pulps and the cavity filled with gutta-percha, oxysulphate or oxyphosphate mixed with an essential oil.

3. The Clowes "safety pockets" have been faithfully tried, and arsenic applied to the dentine and covered up, safe from pressure, and from other possible accidents.

4. Physic has been thrown to the dogs and surgery called in, and the "knocking out" plan, with a sharpened stick, wet with carbolic acid, driven into the straight-rooted teeth, or anæsthetics, notably nitrous oxide, have been administered, and sharp burs revolved by the dental engine have been thrust into the pulp chambers.

The Charles Kingsley method of delicately introducing a watchmaker's broach into the canal to the apex along the side of the pulp and rapid twisting of the broach as a final act has been tried once or twice.

A return to drug methods followed, and nitrate of silver, nitric acid, creasote, oxide of zinc saturated with creasote, as a capping and slow devitalizer, have all been tried.

After thirty years of constant practice I have to confess with shame, that I still have trouble, and by far too large a proportion of failures in this common operation of devitalizing these little organs, so that I may remove them perfectly and painlessly from the teeth and put gutta-percha or some other material in their places.

The necessity for removing these bodies is almost a daily one. In my practice, it is an important and exceedingly common operation in dental surgery. It is performed hundreds of times each year, and yet the proportion of failures to perform it speedily, easily, scientifically, and painlessly is appalling. My idea of a successful operation is that when it is desirable to devitalize and extirpate a pulp we should be able to apply a drug which instead of acting as a violent irritant and causing hyperæmia and intense pain, should benumb permanently the nerve fibrils at once, and cause either atrophic changes in the connective and other tissues of the pulp, or rapid degenerations, so that the entire organ can be lifted out or washed out, after a reasonable time has passed, let us say within a week or a month. Arsenious acid does accomplish devitalization, and degenerative changes follow; but its action is so uncertain as regards time and completeness throughout the organ, and so much pain accompanies it that I feel that something else should be by this time evolved from the inner consciousness of dental therapeutic or materia medica experts. In my hands cobalt behaved so much like arsenious acid that I could not detect any clinical difference. The surgical methods seemed applicable in only a limited number of cases and even where I have resorted to nitrous oxide and the bur, difficulties have arisen in the devitalization of the pulp stumps. In the devitalization of perfectly healthy pulps, which have never been irritated to a hyperæmia beyond the normal, the slow methods of capping with creasote and oxide of zinc, sulphate of zinc, etc., are indicated in certain cases; not, of course, in those cases where we wish to devitalize and extirpate speedily for the special operation of afterwards filling the root canals.

These cases arise in my practice a few times each year, where, after fractures of the crown, malformation of the enamel, etc., crowns of some sort are indicated.

Dr. Frank Abbott is reported as saying, "The only one condition where I think of using any material for devitalizing the pulps of teeth, is where it is impossible to stop pain," and he adds, "I have for the last fifteen years used arsenic in teeth as many as three and four times and no more." Under my hand, where a pulp is fully exposed and where odontalgia ensues on account of exposure, partial or complete, pain can be quieted for a limited period, but in a majority of cases returns at the most inconvenient of times, and these teeth that I have nursed and cared for so tenderly, too frequently present themselves to me in the future, after more thorough operations by some dentist in a neighboring city, or in a summer or winter resort. That is, my operations were temporary and upon a recurrence of pain some other dentist has removed the then devitalized pulp and filled the roots and crowns in a superior manner, leaving me simply the *blush.*

Drs. Witzel, Baume, Herbst, Bodecker and Miller look for succor in the direction of preserving certain portions of the stumps of pulps in the roots of teeth after what might be called a partial devitalization of the coronal portion of the organ. This is, it seems to me, a compromise between the Abbott school and the removal of the last vestige school, and success may lie between these two schools. Certainly the patient experiments of Dr. Miller in this direction are worthy of our highest interest. A curious phenomenon presents itself in the lives and deaths of these pulps of teeth. If, in crossing a heath instead of pitching my foot against a stone or a watch, as the great Paley might have said, I should experience through the cilliated and other epithelia of the regio olfactory in my nasal passages a stimulation or irritation from the emanations of a defunct rodent, would the pschycological inquiry arise, "Does the rat still feel and hear and see and know, even if putrefactive changes appeal loudly to my senses? Yet as dentists, have we not daily evidence that putrefactive changes may be far advanced in a dentinal pulp, and that acute irritability of nervous tissue remains at the same time? The lymphatic, the connective, and other tissues may be reduced to an almost liquid state of decomposition, and yet these nerves which are believed to be trophic in function and can hardly be purely vaso-motor, seem to maintain a watchful care over the corpse of the pulp, and they awaken the guard in the centres of sensation by loud calls for assistance, whenever the dentist, acting as a ghoul or undertaker, attempts to remove the decomposing corpse from the house (or canal) where it formerly lived. This is an indisputable fact, and this is why I beg of materia medica to offer a medicine, the first quality of which shall be to benumb nervous tissue itself.

